# Motion artifacts in capacitive ECG monitoring systems: a review of existing models and reduction techniques

**DOI:** 10.1007/s11517-024-03165-1

**Published:** 2024-07-20

**Authors:** Matin Khalili, Hamid GholamHosseini, Andrew Lowe, Matthew M. Y. Kuo

**Affiliations:** 1 Institute of Biomedical Technologies, Auckland University of Technology, 6 St Paul St, Auckland, 1010 New Zealand; 2 Department of Electrical and Electronic Engineering, Auckland University of Technology, 6 St Paul St, Auckland, 1010 New Zealand; 3 Department of Computer Science and Software Engineering, Auckland University of Technology, 6 St Paul St, Auckland, 1010 New Zealand

**Keywords:** Adaptive filtering, Digital signal processing, ECG capacitive electrodes, Electrode-tissue impedance, Motion artifact

## Abstract

Current research focuses on improving electrocardiogram (ECG) monitoring systems to enable real-time and long-term usage, with a specific focus on facilitating remote monitoring of ECG data. This advancement is crucial for improving cardiovascular health by facilitating early detection and management of cardiovascular disease (CVD). To efficiently meet these demands, user-friendly and comfortable ECG sensors that surpass wet electrodes are essential. This has led to increased interest in ECG capacitive electrodes, which facilitate signal detection without requiring gel preparation or direct conductive contact with the body. This feature makes them suitable for wearables or integrated measurement devices. However, ongoing research is essential as the signals they measure often lack sufficient clinical accuracy due to susceptibility to interferences, particularly Motion Artifacts (MAs). While our primary focus is on studying MAs, we also address other limitations crucial for designing a high Signal-to-Noise Ratio (SNR) circuit and effectively mitigating MAs. The literature on the origins and models of MAs in capacitive electrodes is insufficient, which we aim to address alongside discussing mitigation methods. We bring attention to digital signal processing approaches, especially those using reference signals like Electrode-Tissue Impedance (ETI), as highly promising. Finally, we discuss its challenges, proposed solutions, and offer insights into future research directions.

**Graphical abstract:**

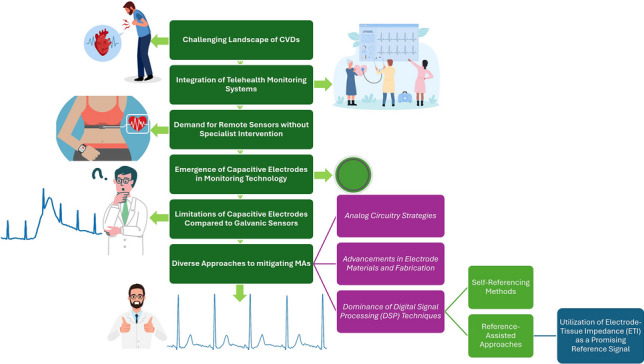

## Introduction

Cardiovascular diseases (CVDs) are a group of disorders affecting the heart and blood vessels. They are a leading cause of death worldwide, claiming more than 17.9 million lives annually [1]. Cardiac arrhythmia significantly contributes to almost 80% of CVD cases [2]. While some arrhythmias are not life-threatening, others, such as atrial fibrillation, premature ventricular contractions, and excessive supraventricular ectopic beats, can result in severe conditions like stroke, cardiac arrest, or heart failure [3]. The early detection of arrhythmias can prevent around 90% of CVDs [4]. To achieve this goal, physicians extensively employ electrocardiogram (ECG) technology to identify abnormal heart rhythms, investigate the origins of chest pains, and categorize CVDs in clinical settings. Typically, an ECG signal comprises P waves, QRS complexes, and T waves [5–7], with any variations in these parameters indicating potential abnormalities in heart function. The frequency range of ECG signals generally spans from 0.67 to 120 Hz [6], although the literature often reports deviations from this range depending on the intended ECG application and the risk profile of the studied population [8, 9]. Additionally, real-time and long-term ECG monitoring has become important, serving two crucial purposes. Firstly, timely emergency care within the 'Golden Hour'—the first hour following the onset of cardiac symptoms—can significantly reduce mortality and improve the quality of life for patients with specific heart conditions, such as cardiac arrest [5, 10]. Secondly, continuous disease monitoring facilitates ongoing health management by effectively treating patients with certain types of CVDs. These conditions require extended monitoring periods to enhance disease diagnosis and prescribe the most effective treatment for the patients. However, the excessive cost of medical treatment poses a significant challenge, prompting a shift from hospital-centred to patient-centred healthcare [11]. This shift has led to the development of intelligent solutions and telemedicine applications [12]. This offers two distinct advantages: reducing patients' reliance on hospitals and lowering expenses. Moreover, telemedicine can improve public health, particularly in ageing societies in developed countries and in low- and middle-income areas. Presently, many studies are exploring the possibility of virtual care for people living in rural areas affected by CVDs [13]. After the COVID-19 pandemic, the importance of telemedicine has further grown. Notably, researchers such as Jiang et al. [14] have proposed wearable telehealth solutions for monitoring essential physiological parameters in COVID-19 patients, such as heart rate, blood oxygen saturation, and respiratory rate.

Telemedicine should provide patients with independent remote sensors, ensuring safety, comfort, precision, and real-time long-term monitoring capabilities without the need for specialized assistance. Traditional gel/wet ECG electrodes (Ag/AgCl), extensively used in clinical and research settings, are not useful in this field because these electrodes require direct skin contact through an electrolytic gel applied by technicians [12, 15–20]. They need multiple disposable electrodes, frequent need to be replaced, and there is significant potential for skin irritation. Consequently, they are not suitable for prolonged monitoring or use outside of a hospital setting [16, 17, 19, 21–25]. To address the limitations of conventional ECG measurement methods, capacitive electrodes were introduced as an innovative approach to measure skin surface potentials without necessitating direct conductive coupling to the skin, allowing measurement through materials such as hair, cloth, insulation layer, or air [26]. Their versatility has led to integration into numerous personal healthcare applications, including furniture, exercise aids, automotive devices, and wearable devices [17]. This innovation offers a cost-effective, long-term diagnostic solution for personal healthcare monitoring that avoids skin preparation and irritation [21, 27, 28]. Using these sensors can embed healthcare monitoring into patients' daily lives and improve the health condition of people who have not yet developed a disease [29]. Moreover, they play a key role in monitoring heart activity beyond clinical settings in ambulatory ECG measurement systems. In contrast to traditional ECG systems that utilize ten electrodes, ambulatory ECG systems are characterized by their compactness, portability, and reliance, typically, on only 2–3 electrodes [25]. However, the design of electrodes involves finding a balance between comfort and reliable monitoring [21] and ensuring comparable quality to traditional medical instruments remains paramount. Despite the availability of heart rate monitoring sensors for consumers as standalone devices and integrated into smart technology, the challenge persists in achieving diagnostic precision comparable to hospital-grade 12-lead systems [30, 31]. Some of these challenges is because of some limitations in capacitive ECG electrode front-end. Moreover, there are several types of interference that can potentially contaminate the ECG signal in capacitive electrodes, including electromagnetic interference (EMI) and motion artifacts (MAs). EMI, particularly power-line interference (PLI), consistently introduces significant common-mode (CM) noise. PLI, with a frequency of 50 Hz in Europe and 60 Hz in the United States, is a major noise source in any ECG measurement system and is especially evident in capacitive ECG measurements. Generally, PLI is primarily caused by displacement currents coupling either into the measurement system or into the subject's body, and it appears in the final recordings due to unbalanced electrode impedance. However, it can be reduced to some extent by optimizing front-end electronics and system design [21, 32, 33].On the other hand, the unidentified interference is commonly referred to as MA while its exact origin, cause, and definition remain unclear. MAs are commonly explained by the fluctuating high impedance between the body and the electrode because of body movements [21]. The variation of this impedance can modulate circuit leakage potentials and leads to MA. Another potential cause of MA is the triboelectric potential, which arises from the friction and separation between the electrode and the body. These interferences need to be mitigated to increase the SNR and ensure the reliability of the ECG signal for interpreting different types of cardiovascular diseases (CVDs) without misdiagnosis.

In many studies on capacitive ECG electrodes, MAs and their impact on the ECG signal are recognized as significant challenges, prompting numerous investigations to address these issues. Despite the proven adverse effects of MAs in ECG recording systems, their origins remain poorly understood and subject to varying interpretations across different research studies. However, these interpretations can vary due to differences in analog designs, electrode materials, and environmental conditions such as temperature and humidity, all of which uniquely influence system performance. A notable gap exists where these varied definitions and interpretations have not been consolidated into a single source for comparative analysis. To address this gap, we provide a detailed overview of MAs, encompassing their origins, effects on ECG signals, and contributing factors based on existing literature, to enhance the understanding of MAs.

We proceed by reviewing the latest techniques for mitigating MAs, focusing particularly on Digital Signal Processing (DSP) methods. Specifically, we emphasize reference-assisted techniques using Electrode-Tissue Impedance (ETI) as a reference, as studies suggest that the variable high impedance between the body and electrodes during motion significantly contributes to MAs. While this method shows potential, there is a lack of thorough reviews in the existing literature, which we intend to address. To facilitate discussion, we present a comparative table summarizing previous works. However, comparing their efficiencies directly poses challenges due to variations in R&D statuses, design approaches, experimental methodologies, and selected performance metrics among others. Our primary objective is to explore methodologies used by each study, the challenges encountered, approaches taken to address them, and the success of their techniques. Finally, based on these reviews, we outline future research directions for those continuing work in this area.

However, addressing MAs is not the sole focus of our review. A complete understanding of capacitive ECG systems is crucial for identifying their causes and distinguishing them from other interferences. Therefore, optimizing signal quality by mitigating non-motion-related interferences precedes directly addressing MAs. To start, we discuss the fundamentals of capacitive ECG systems, which include discussing the limitations of capacitive electrodes and the impact of CM interferences on signal quality. Following this, we investigate strategies for enhancing front-end electronics and techniques aimed at improving the CMRR throughout the entire system.

Therefore, this article is structured as follows: Section 2 introduces the basics of capacitive ECG measurement systems. Section 3 addresses drawbacks in capacitive electrode circuitry and discusses solutions to improve the signal quality. Section 4 discusses the defined concept of MA in the literature, its origins, and its impact on the ECG signal. Current techniques for reducing MAs are addressed in Section 5. This part is divided into three key domains: analog circuits, electrode materials and fabrication, and DSP techniques. These domains must be addressed in parallel to achieve optimal results. Notably, this article emphasizes the DSP domain as a principal technique for effectively mitigating MAs. Section 6 engages in a discussion of the presented techniques and outlines potential directions for future research. Finally, Section 7 concludes the paper.

## Capacitive ECG measurement systems

In capacitive ECG sensors, an interface between skin and electrode transfers the ionic current in human body into biopotentials that can be measured by a signal acquisition circuit [21]. Before exploring the specifics of capacitive ECG measurement systems, it's crucial to address the electrode-body interface for capacitive electrodes.

### Electrode–body interface for capacitive electrodes

**Fig. 1:**
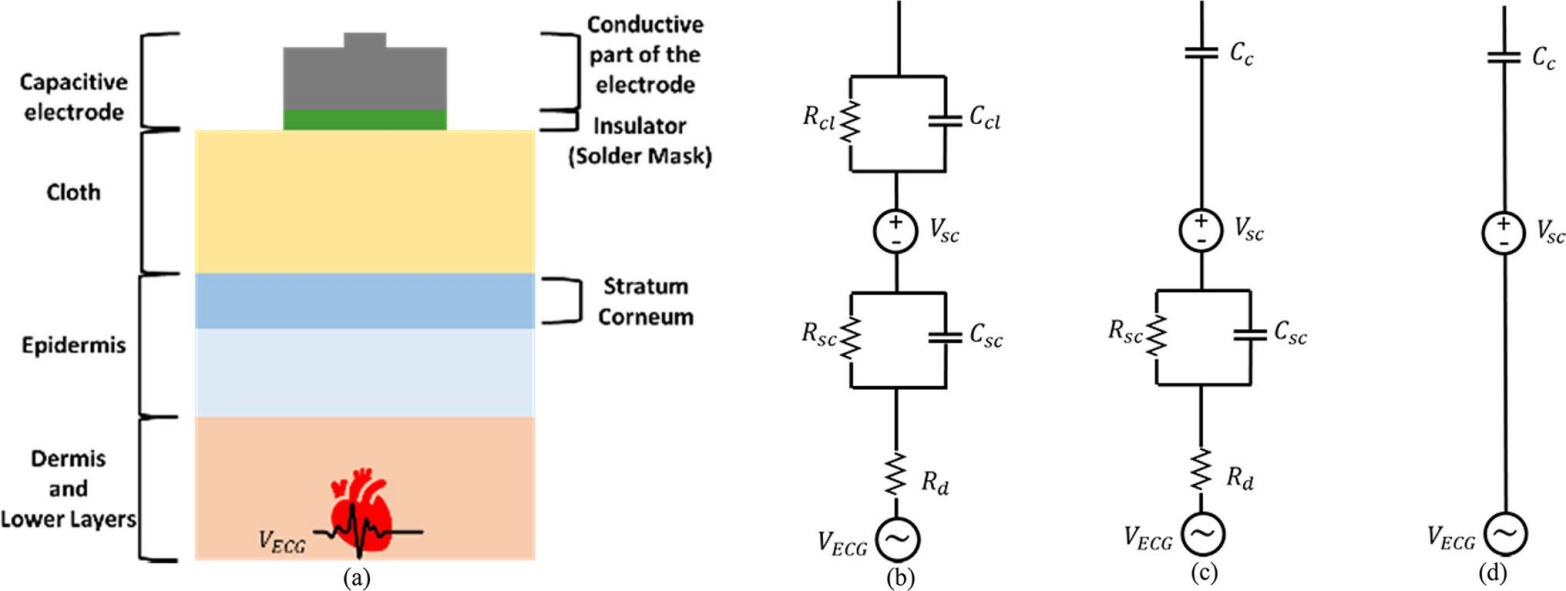
Illustrations of the electrode–body interface for capacitive electrodes. **a** General layer stack. **b** General equivalent circuit model. **c** Equivalent circuit model without cloth (Representation of direct contact between electrode and skin). **d** Simplified circuit model without considering cloth

Biopotential sensing utilizes electrodes to acquire signals from the body. Often, these electrodes are not in direct contact with body fluids; instead, they reside on the surface of the skin, known as surface electrodes [21, 34]. The characteristics of the electrode–body interface for surface electrodes depend on the skin’s electrical properties and the type of electrode [34]. In general, the coupling between the skin and electrode can be described as a layered conductive and/or capacitive structure, with series combinations of parallel R and/or C elements. The general layer stack and equivalent circuit model of capacitive electrodes coupled with the skin, based on various models found in the literature, result in structures depicted in Fig. 1a and b respectively.

The skin consists of three primary layers: the epidermis, dermis, and subcutaneous layer. The inner layers, dermis, and subcutaneous layer are highly conductive and are in direct contact with inner bodily fluids rich in ions, which is equivalent to the resistance ${R}_{d}$ [34, 35]. In the epidermis layer, the Stratum Corneum, a high-resistance layer of the skin, is dominant, considered to have a resistance ${R}_{sc}$ and capacitance ${C}_{sc}$ in parallel [35–37]. The cloth layer is considered as a resistance ${R}_{Cl}$ in parallel with a capacitor ${C}_{cl}$, where ${R}_{Cl}$ is the resistance of the fabric surface and ${C}_{cl}$ is the skin–electrode capacitance. This coupling impedance in the body–sensor interface is primarily influenced by the subject's clothing in this scenario [35, 38].

Since there is no electrode–electrolyte interface present, the circuit model of capacitive electrodes excludes impedances associated with this interface, as well as the electrode–electrolyte potential at equilibrium, referred to as the half-cell potential, ${V}_{HC}$, which is characteristic of wet electrodes. However, in numerous papers, the skin potential, ${V}_{sc}$, is considered for these electrodes [32, 34, 39]. The stratum corneum acts as a semipermeable membrane, effectively separating electrolytes, sweat, and body fluids of varying ionic concentrations. Consequently, this arrangement creates a potential difference across the stratum corneum, referred to as the skin potential [34]. It is worth noting, though, that in many papers, this potential is not accounted for in the context of capacitive electrodes.

Moreover, in some papers, the cloth layer is omitted, indicating that the capacitive electrode is directly connected to the skin, as shown in Fig. 1c. In this scenario, ${C}_{c}$ is considered to model the insulation layer between the electrode and the skin surface, and no resistor is taken into account [34, 36, 37].

In numerous articles where the electrode directly interfaces with the body, there's an assumption that ${C}_{c}$ represents the dominant layer in capacitive electrodes, thereby disregarding the impedance of other layers. This assumption stems from the fact that the value of this capacitor is typically lower compared to the other layers. Conversely, if the impedance of this layer is higher than that of the others, it leads to the equivalent model illustrated in Fig. 1d [17, 32, 39, 40].

### Equivalent circuit of the capacitive measurement system

**Fig. 2:**
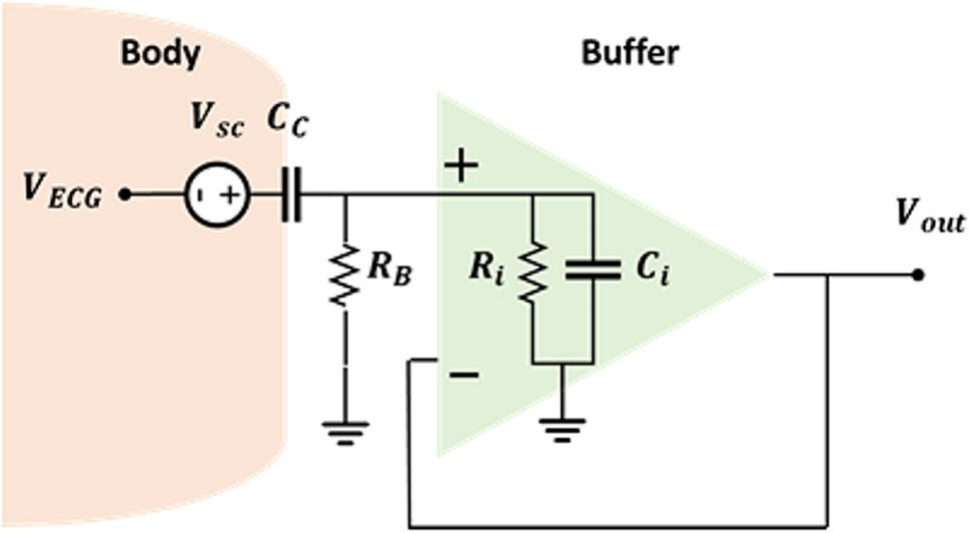
Voltage follower schematic

**Fig. 3:**
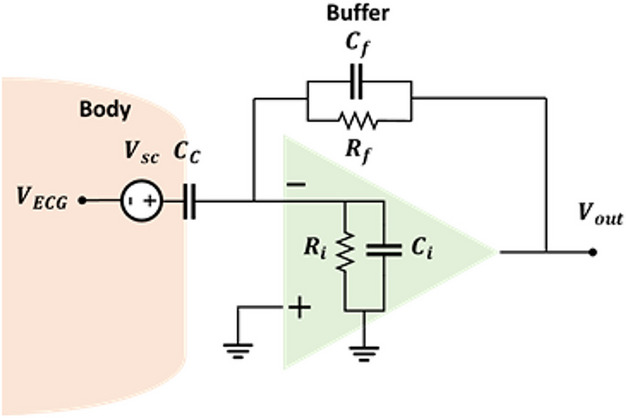
Charge amplifier schematic

After introducing the electrode-body interface for capacitive electrodes, the equivalent circuit of the capacitive measurement system should be introduced. For simplicity, the capacitive electrode, along with the body surface, can be modelled by a coupling capacitor ${C}_{c}$, in series with the skin potential ${V}_{sc}$, as shown in Fig. 1d. In practice, ${C}_{c}$ can be as small as a few pF, depending on factors like electrode size, dielectric material, and material thickness. Typically, an active electrode is used as an ECG acquisition system, with the front-end circuit including a buffer, usually an OpAmp with an ultra-high input resistor ${R}_{i}$ and a low input capacitor ${C}_{i}$, along with active shielding. This setup enables transmission over long cables and mitigates sensitivity to PLI and cable movement.[21]. This can be achieved using a voltage follower/amplifier (VA) or a charge amplifier (CA), depicted in Figs. 2 and 3, respectively. Other circuits like Instrumentation Amplifiers (INAs), bootstrap circuits, or application-specific integrated circuits (ASICs) with high input impedance [41] are also mentioned in the literature, designed for high input impedance. The VA, a noninverting amplifier, is preferred for the front-end in most cases due to its relatively high input impedance, resulting in less signal attenuation compared to CA [21]. Conversely, CA is rarely employed for capacitive biopotential recording [42, 43] although some studies have reported successful ECG recordings using a CA [42]. Researchers like Peng et al. [44] have compared VA and CA designs in terms of their common mode rejection ratio (CMRR), noise performance, and frequency response. Xiao et al. [45] extended this comparison to their responses to artifacts caused by motion or interference. Their experiments indicated that the CA performs better under conditions of poor coupling (small capacitance, of the order of a few pF) due to its linear gain response and quicker recovery from triboelectricity artifacts. On the other hand, the VA performs better under good coupling conditions (hundreds of pF) due to its improved CMRR, stable gain, low noise, and reduced susceptibility to ${C}_{c}$ variation artifacts. These two interferences are multiplicative in a VA, but remain independent in a CA. Consequently, a VA is preferred when triboelectricity artifacts are low and coupling capacitance is high whereas a CA is preferred when these artifacts are high, due to its faster recovery time [41, 44, 45]. However, overall, the VA is more widely adopted and accepted, and this paper exclusively focuses on investigating the VA.

## Limitations in capacitive ECG measurement circuits

**Fig. 4:**
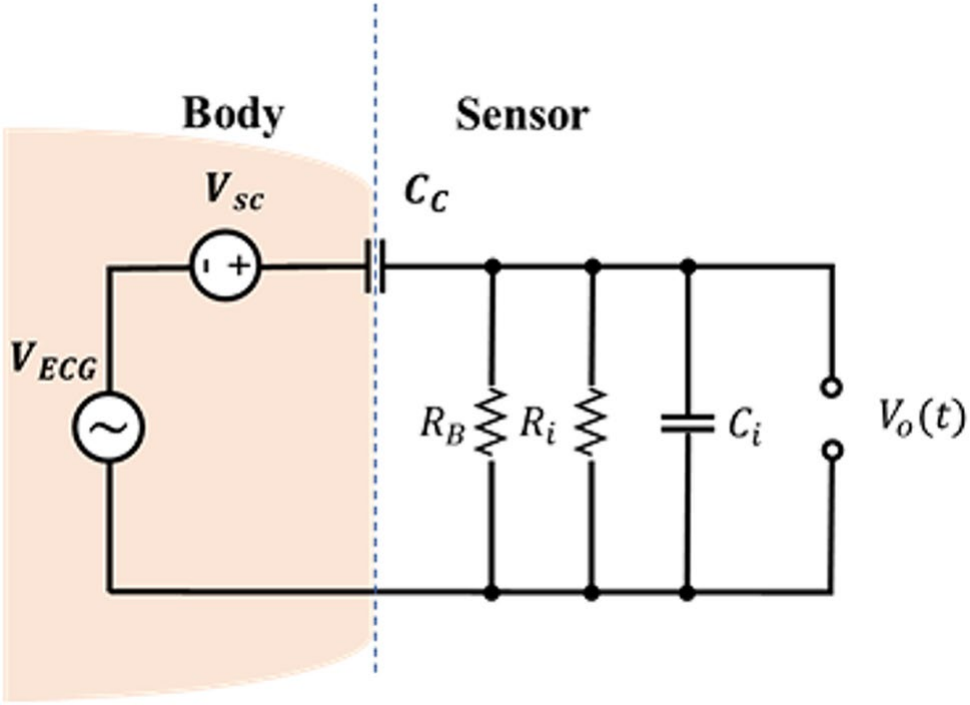
Equivalent circuit model of the capacitive measurement system

The electrical equivalent model shown in Fig. 2 is depicted in Fig. 4. It consists of the capacitor formed between the electrode's plate and the body $\left(C_c\right)$, the skin potential source $\left(V_{sc}\right)$, and the ECG signal represented as ${V}_{ECG}$. The front-end circuit includes the input resistor $\left(R_i\right)$ and capacitor $\left(C_i\right)$ of the OpAmp. Additionally, the bias resistor $\left(R_B\right)$ is typically used with this configuration. In the subsequent discussion, we study the mentioned parameters and the difficulties encountered during the circuit design. However, it should be noted that due to the significant impact of ${V}_{sc}$​ on the circuit, it will be discussed later in Section 4.

### Bias resistor

Ideally, op-amps would exhibit zero bias current. However, practically, bias current exists, and its magnitude varies depending on the type of op-amp. To address this issue, a bias current path can be used by connecting a resistor to the ground (refer to Fig. 4). This path is essential since bias current cannot flow through the human body and the charge will accumulate at the input front end [21, 28]. While a bias resistor is needed to discharge the input of the amplifier from its input bias current, it can introduce other problems. In this type of measurement, achieving a large input resistance is desirable, often by selecting an op-amp with a large input resistance, typically within the Tera Ohm range [21]. However, placing the bias resistor in parallel with the op-amp's input resistance can significantly reduce the overall input resistance. To overcome this, using a high-value resistor is a solution, although this can introduce issues related to drift and thermal noise. Moreover, it is difficult to fabricate tera-Ohm off-chip resistors. Additionally, using a bias resistor can cause an offset in the circuit. Though the induced offset voltage can be relatively high, it can be removed by filtering the dc component or by using negative feedback [46, 47]. Another consideration is the potential for op-amp saturation. In such cases, the maximum value of the resistor should be lower than $\frac{{V}_{cc}}{{i}_{Bias}}$ for rail-to-rail input stages [48]. Some researchers have tried including reset switches to manage saturation [45, 49, 50]. Moreover, Moreno-García et al. [17] found that the op-amp they employed, LMP7721, had a sufficiently low leakage current. In this case, they did not use a bias resistor in their design. They employed a passive RC high-pass filter to eliminate the generated offset. There are alternative approaches to establishing the bias path without relying solely on high-value resistor, for instance, the reverse leakage current of a low leakage silicon diode [36, 41], or bootstrap circuits [51–53]. However, implementing these circuits can cause more complexity and pose new challenges.

### Frequency response of front-end circuit

A high-pass filter is formed in the front-end circuit due to the presence of ${C}_{c}$ and the input impedance of the buffer. The input resistance of the circuit is determined by ${R}_{i}||{R}_{B}$. Since ${R}_{i}$ (the input impedance of the op-amp) is significantly larger than ${R}_{B}$, the total input resistance can be approximated as ${R}_{B}$.

The frequency response of this filter is calculated using Eq. (1). The magnitude of the filter can be obtained from Eq. (2). By considering a very high bias resistor, this equation can be simplified to (3). The cut-off frequency of the filter can be obtained from Eq. (4).

1 $$H\left(j\omega \right)=\frac{{R}_{B}{C}_{c}\omega j}{1+{R}_{B}\omega j\left({C}_{c}+{C}_{\text{i}}\right)}$$

2 $$\left|H\left(j\omega \right)\right|=\frac{{R}_{B}{C}_{\text{c}}\omega }{\sqrt{1+{\left({R}_{B}\omega j\left({C}_{\text{c}}+{C}_{\text{i}}\right)\right)}^{2}}}$$

3 $$\left|H\left(j\omega \right)\right|=\frac{{C}_{\text{c}}}{{C}_{\text{i}}+{C}_{\text{c}}}$$

4 $${f}_{c}=\frac{1}{2\pi {R}_{B}\left({C}_{\text{c}}{+C}_{\text{i}}\right)}$$

It is important that the cut-off frequency of the formed high pass filter, ${f}_{c}$,be low enough to allow the lower ECG frequency components to pass through [28]. The question arises: how low should this cut-off frequency be? As previously mentioned regarding the ECG frequency range, the answer depends significantly on the intended application and the risk profile of the studied population. This underscores the importance of understanding the significant information gained or lost by selecting a specific frequency range. To address this concern, Young [9] published the proposed frequency requirements for the 80,601–2-86 committee draft in 2019, which can be found in Table 1, providing general guidance on selecting the ECG frequency range. Consequently, deviations can be observed in papers discussing capacitive measurement systems. For instance, Sun and Yu [21] proposed an ECG range starting from 0.05 Hz to preserve complete signal information, particularly emphasizing ST measurements shown in Table 1. In contrast, Torfs et al. [40] have suggested a cut-off frequency of 0.67 Hz for ambulatory ECG measurements, while, Asl et al. [28] recommend a cut-off frequency of 0.1 Hz, citing alignment with commonly used bandpass filters in ECG analysis.

**Table 1:** Summary of proposed frequency requirements in the 80,601–2-86 committee draft [9]

Intended use	Frequency response requirement
General use (rhythm interpretation)	0.67–40 Hz for all populations
ST measurement	Meets rectangular or triangle impulse response (0.05 Hz IIR or 0.67 Hz ZPD filter)
Diagnostic ECG (contour interpretation)	0.67–150 Hz for all populations and meets ST monitoring requirement

After determining the value of ${f}_{c}$, the remaining parameters in Eq. (4) can be calculated. The value of ${C}_{\text{c}}$ varies based on factors like sensor size, material, distance from the body, and insulator material. For instance, it may be as low as 0.1 pF when the signals are picked up through clothes and an air gap at a distance of > 100 mm. However, when the sensor is tightly attached to a cotton layer, the value be around 30 pF.

Furthermore, the input capacitance of op-amps, ${C}_{\text{i}}$, has remained relatively constant over the past decades, typically ranging from 3-10pF [54]. As mentioned earlier, ${R}_{i}$ is almost equal to ${R}_{B}$. Based on Table 1 for general usage and adopting $f_c=0.67$, and $C_\text{c}=30\mathrm{pF}$ , using Eq. (4) allows us to deduce ${R}_{B}$, yielding a minimum value of 7 GΩ.

Ideally, the front-end circuit should act as a buffer with a flat frequency response. However, due to the voltage divider formed by ${C}_{\text{c}}$ and ${C}_{\text{i}}$ in Eq. (2), there may be some signal attenuation [17, 55]. The ECG signal voltage typically ranges from 1 to 5 mV. Therefore, even slight attenuation in the front end can decrease SNR, leading to a lower quality signal. Therefore, selecting an amplifier with a very small input capacitor is essential to minimize this attenuation. If ${C}_{\text{i}}$ is sufficiently low, and the electrode, firmly attached to the body with a large area, results in a high ${C}_{\text{c}}$ there should be no problem. But in situations where the distance between the body and the electrode increases, leading to a decrease in ${C}_{\text{c}}$, difficulties may arise. To address this issue,

techniques like power supply bootstrapping have been used over the years to reduce input capacitance [54, 56–58].

In capacitive ECG measurement, the electrodes are not fixed to the body, and even if they are tightly fastened, some movement remains. Additionally, the body’s natural physiological vibrations can impact the coupling capacitance, even when no deliberate or unintended body movements occur [38]. These physiological vibrations include respiration and cardiac contraction, which can alter this coupling capacitance [59]. While many studies have overlooked this issue, Uguz et al. [38] studied the mechanical vibrations of the human body itself as a source of time-variant coupling capacitances. According to Eq. (5), changes in the front-end capacitor value are due to the varying distance between the electrode and the subject's body. However, the dielectric constant $\left({\mathcal E}_r\right)$ and the effective area of the electrode surface (A) remains constant.

5 $${C}_{c}\left(t\right)=\frac{{\mathcal{E}}_{r}A}{d\left(t\right)}$$

In this case, we are not dealing with a linear time-invariant (LTI) system due to the varying component ${C}_{c}(t)$. Even if we approximate the system as LTI within short intervals with a constant ${C}_{c}$, Eqs. (1) to (4) will remain time-variant due to this changing ${C}_{c}$. Consequently, both ${f}_{c}$ and $\left|H\left(j\omega \right)\right|$ exhibit time-variant values.

**Fig. 5:**
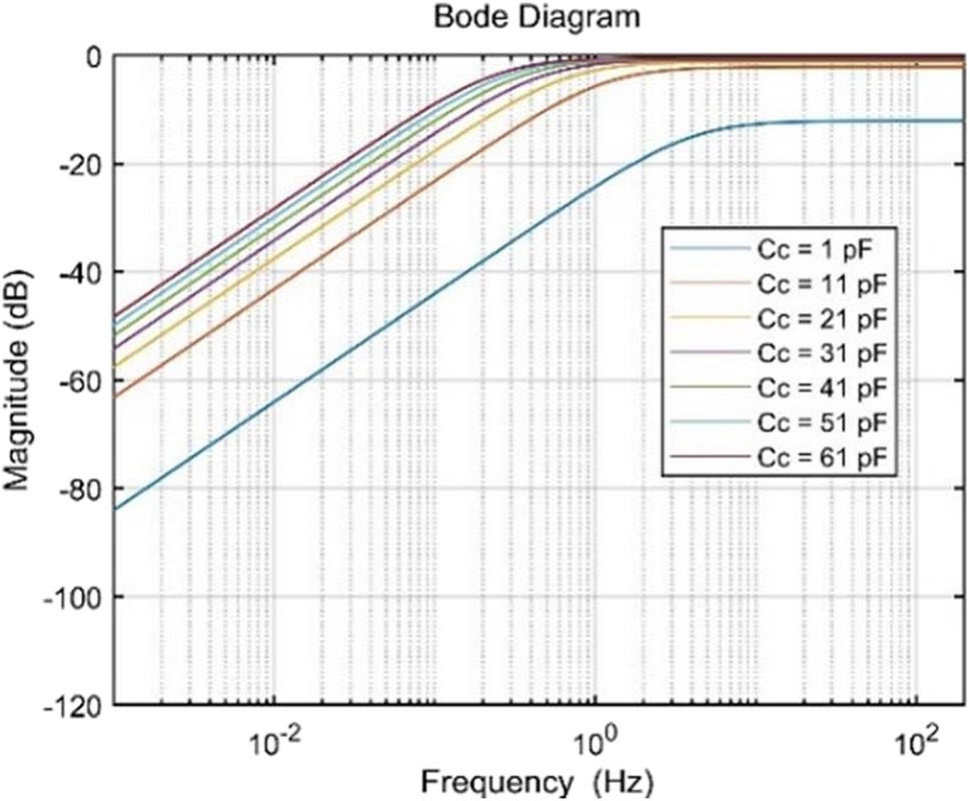
Front-end circuit Frequency response for different values of ${C}_{c}$

For the specific values $R_B=10\mathrm G\Omega$, $C_\text{i}=3\mathrm{pF}$, along with seven different ${C}_{c}$ values, Fig. 5 illustrates the Bode diagram of the circuit. Notably, when $C_c=1\mathrm{pF}$, $\left|H\left(j\omega \right)\right|$ is -12 dB, considerably lower than 0 dB. As ${C}_{c}$ increases, $\left|H\left(j\omega \right)\right|$ approaches 0 dB. Simultaneously, the rise in ${C}_{c}$, leads to a decrease in ${f}_{c}$, aligning with the desired outcome. Essentially, the system behaves like a dynamically changing high-pass filter for the ECG signal, ${v}_{ecg}\left(t\right)$ [39]. In simpler terms, the time-varying coupling impedance causes multiplicative distortion of the ECG signal, resulting in a distorted version of ${v}_{ecg}\left(t\right)$.

### Challenges of reduced CMRR and solutions

**Fig. 6:**
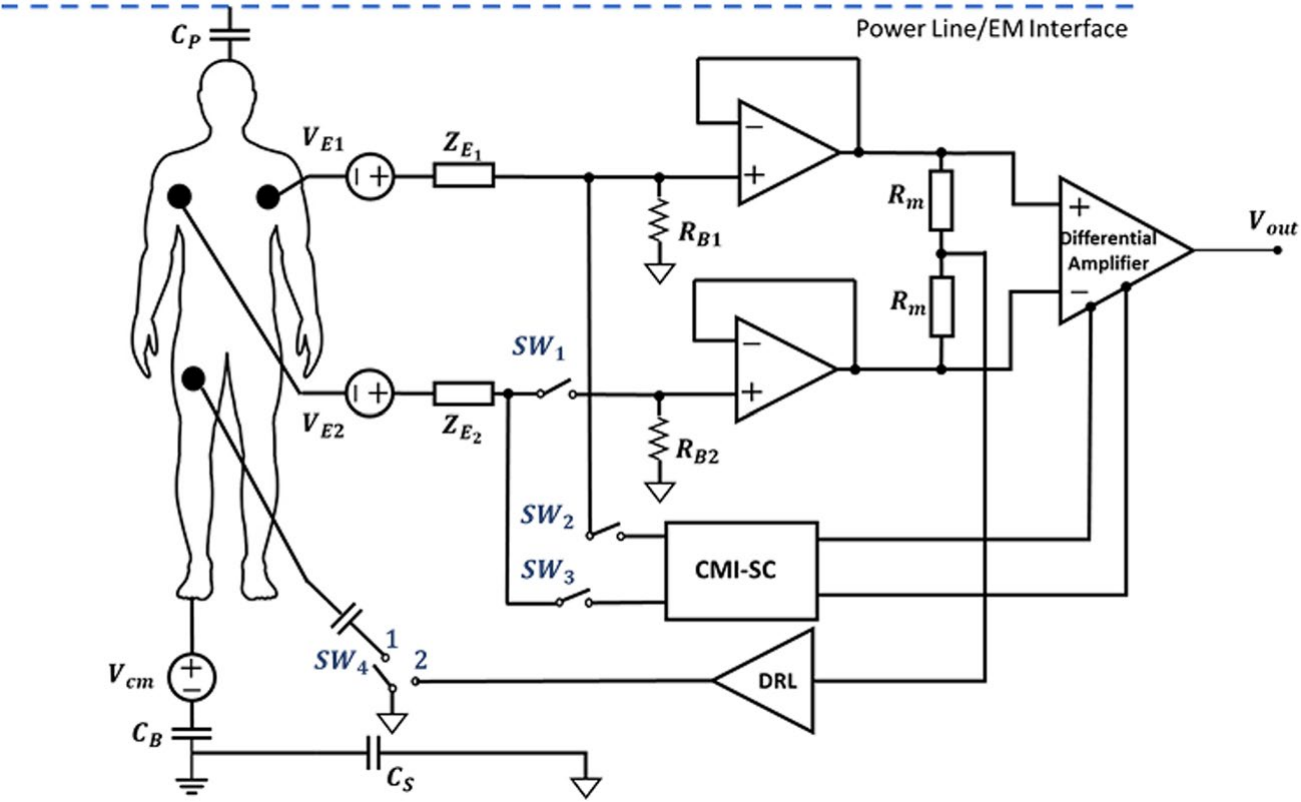
General integrated electrical model for ECG capacitive recording systems

In this section, we introduce a comprehensive integrated electrical model for ECG capacitive recording systems, as depicted in Fig. 6. Within this model, there are non-common mode voltages denoted as ${V}_{E1}$ and ${V}_{E2}$ which are specific to each electrode and thus cannot be rejected using the differential measurement method and will be addressed later in the text. These individual voltages stem from various sources such as skin potential (${V}_{sc}$), as discussed in Section 2.1, or other sources. The primary focus of this section is on CM noises, which are typically mitigated using the differential measurement technique in many applications.

In capacitive measurement systems, the body is electrically isolated, and this floating state makes it highly susceptible to external noises, thereby affecting the measurement system, particularly the CM noise [60]. The presence of CM noise sources, such as power lines, EMI, or wireless power transfer systems, can significantly disrupt the accurate detection of signals [15]. In an ECG system, considerable EMI can couple to the patient's body through the skin. Additionally, EMI may couple to the ECG system through coupling capacitors ${C}_{P}$ and ${C}_{B}$, as illustrated in Fig. 6. Moreover, it can also be introduced to the system through the long ECG signal measuring cables and protection circuitry, typically located at the front of the ECG system. Furthermore, capacitance ${C}_{S}$ is coupled between the AC ground and the ground of the ECG subsystem. The entire system's CMRR is dependent on the capacitance value of ${C}_{S}$ [61]. The voltage source ${V}_{cm}$ represents other CM voltages that are detectable throughout the body. The values of capacitors and ${V}_{cm}$ are indicative and may vary depending on the measurement methodology, environmental conditions, and parameters of the measuring device.

In general, the differential measurement method is highly effective in reducing CM noise due to its high input impedance and CMRR [62]. However, in capacitive ECG measurement systems, this method does not perform well because of the large asymmetry in the two signal paths. This asymmetry is caused by the difference in impedances between the two electrodes (${Z}_{E1}$ and ${Z}_{E2}$ in Fig. 6). The capacitances between the body and the capacitive electrodes can differ due to body motion, leading to various amplitudes and phases of noise signals at the input of the differential amplifier. In this scenario, the external noise cannot be considered as the CM signal for the differential amplifier, as they are not the same in each path. Consequently, despite the high CMRR of an INA (around 120 dB), the amplifier's overall CMRR is decreased [52, 60, 63, 64]. Equation (6) shows the differential voltage resulting from the asymmetry in ${Z}_{E}$ proportional to the CM potentials, ${V}_{CM}$. $\Delta {Z}_{E}$ stands for the difference of two electrode–skin impedances and ${Z}_{in}$ represents the mean value of input impedances of the OpAmps $\left(Z_{in1}andZ_{in2}\right)$ [52, 63, 64].

6 $${V}_{diff}\approx {V}_{CM}\frac{\Delta {Z}_{E}}{{Z}_{in}}$$

Referring to Eq. (6), to enhance interference suppression, it is crucial to minimize the difference of impedance $\left(\Delta Z_E\right)$ or reduce the CM voltage $\left(V_{CM}\right)$ as much as possible, or alternatively, increase the ${Z}_{in}$. In the literature, there are four known methods to improve the CMRR of the system. These methods are:

1. Active guarding
2. Isolation Capacitance
3. Driven-Right-Leg circuit (DRL)
4. Common-Mode Interference Suppression Blocks (CMI-SC).

By employing these techniques, the system's ability to reject CM interference can be significantly enhanced, resulting in better overall performance.

#### Active guarding

To mitigate the adverse impact of increased stray parasitic capacitance, a commonly employed technique is known as "guarding". This method involves creating a guarding layer that encircles the input channel and maintains the same potential as the input itself. The primary purpose of this guarding network is to minimize any voltage difference between the input wire and the guarding layer. It can effectively eliminate any parasitic capacitance that may exist between them and safeguard high-impedance sensors from environmental EMI [65, 66].

#### Isolation capacitance

Improving the isolation between the device ground and the patient ground leads to a decrease in ${C}_{S}$, which, in turn, helps to enhance the system CMRR. This improvement is particularly noticeable in portable instruments that operate on batteries, where the CMRR tends to be exceptionally high [61, 67].

#### Driven-Right-Leg (DRL) circuit

In the DRL circuit, the reference electrode plays a crucial role by actively controlling the body's potential through a feedback mechanism. The primary objective is to maintain the body's potential close to a known bias point of the device, which is typically set to the desired CM level of the AFE. This feedback loop helps suppress the body's potential swing with respect to the AFE's ground, effectively reducing the CM input swing into the AFE. The DRL electrode is responsible for sensing the CM voltage on the body. By doing so, it can provide essential feedback to the patient's body, leading to a reduction in the ${V}_{CM}$ as indicated in Eq. (6). Although the actual pathway of the current fed to the body is unknown, it is likely to be more controlled than the pathway taken by the measurement system itself. This process contributes to enhancing the overall performance and stability of the circuit, particularly by improving the CMRR.

#### Common-mode interference suppression blocks

In specific designs, the methods mentioned earlier are not employed. Instead, they rely solely on two sensing electrodes along with a CMI-SC Block in a feedback configuration to effectively reduce CM noises [68, 69]. However, in the absence of a third electrode, a two-electrode ECG AFE results in the grounding potential being virtually isolated from the CM potential on the body. This leads to a difference between the body's CM potential and the device's grounding, resulting in significant CM input. This significant CM input can saturate the differential amplifier, particularly when operating under low-voltage power supply conditions, rendering it unable to accurately reconstruct the ECG signal. Therefore, a critical aspect in preventing system saturation is to limit the CM input range. This is where the CMI-SC Block comes into play, as it detects the CM voltage from an internal node within the AFE and generates a proportional CM suppression signal. By implementing this mechanism, the circuit effectively reduces CM fluctuations at the AFE's input pads through a feedback loop.

By considering all the mentioned techniques, various systems and applications utilize different recording setups, such as single, two, or three electrodes, to measure ECG signals. The general setup is illustrated in Fig. 6, which can be modified using switches $S{W}_{1}-S{W}_{4}$, as detailed in Table 2.

**Table 2:** Various configurations of measurement systems

No	Type	$S{W}_{1}$	$S{W}_{2}$	$S{W}_{3}$	$S{W}_{4}$
1	One active electrode + One ground electrode as reference	Open	Open	Open	Connected to “1”
2	Two active electrodes	Close	Open	Open	Open
3	Two active electrodes + CMI-SC	Close	Close	Close	Open
4	Two active electrodes + One ground electrode as reference	Close	Open	Open	Connected to “1”
5	Two active electrodes + One DRL electrode as reference	Close	Open	Open	Connected to “2”

The first model employs a single electrode for ECG signal collection and another as a ground reference, as demonstrated by Serteyn et al. [39]. Their setup includes an active guard to eliminate CM noises effectively.

In the second model, two electrodes measure the biosignal without a reference electrode, a technique known as standard bipolar capacitive measurement [70]. In this configuration, a differential amplifier or INA is utilized to provide gain and convert the differential signal to a single-ended signal. In this configuration, the absence of any techniques aimed at enhancing the overall CMRR may result in a suboptimal signal quality. However, Yong et al. [71] devised an ECG measurement setup in a bathtub employing only two electrodes and made efforts to minimize PLI through the use of narrow-notch filters. In contrast, many other designs employing a two-electrode setup focus on integrating a CMI-SC circuit, representing the third model [68, 69, 72],to reduce CM noises and improve the SNR of the output signal.

However, many of these designs opt for a more conventional approach found in the literature, utilizing a three-electrode setup. This includes two active electrodes for sensing ECG and one passive electrode, forming the fourth model in Table 2. Alternatively, some designs employ a DRL active electrode as the reference, constituting the fifth model. This choice is made with the aim of enhancing the overall CMRR.

## The source of Motion Artifacts (MAs) and their impact on the ECG signal

The primary concern of capacitive measurement systems is the presence of MAs. In numerous studies, any disturbance in the output signal caused by body movements is termed MA [30, 32, 33, 73, 74]. Such body movements involve respiratory or cardiac activity, as well as voluntary body motions. On the other hand, other studies interpret MAs as transient (but not step) random baseline wander resulting from either patient or electrode movement. These studies establish a separate category for baseline wander noise caused by respiration and electromyography noise arising from muscular movements [24, 75]. Breathing-induced MAs lead to baseline wander appearing in the low-pass filtered signal, making R-peak detection challenging. However, the baseline wander resulting from body movement is significantly more substantial when compared to that caused by breathing alone [32]. Furthermore, MA extends the necessary dynamic range (DR) of input for recording devices, often requiring existing systems to employ low amplification gain in their signal amplification stages [74].

**Fig. 7:**
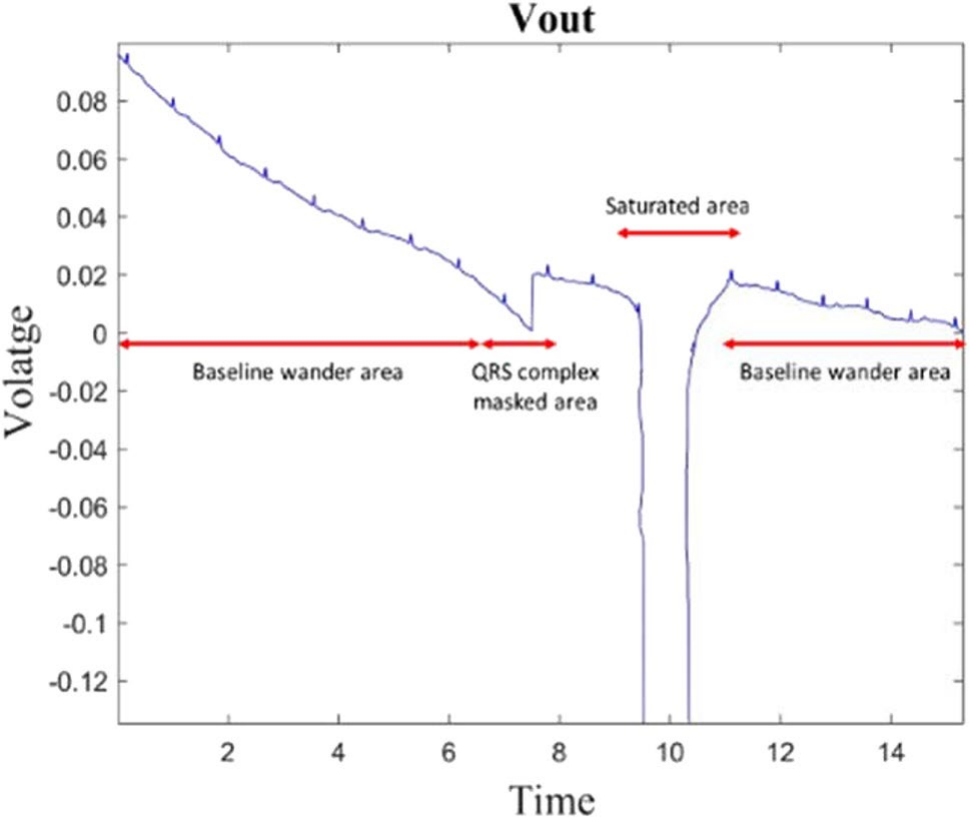
Example of a capacitive ECG signal with body movements

**Fig. 8:**
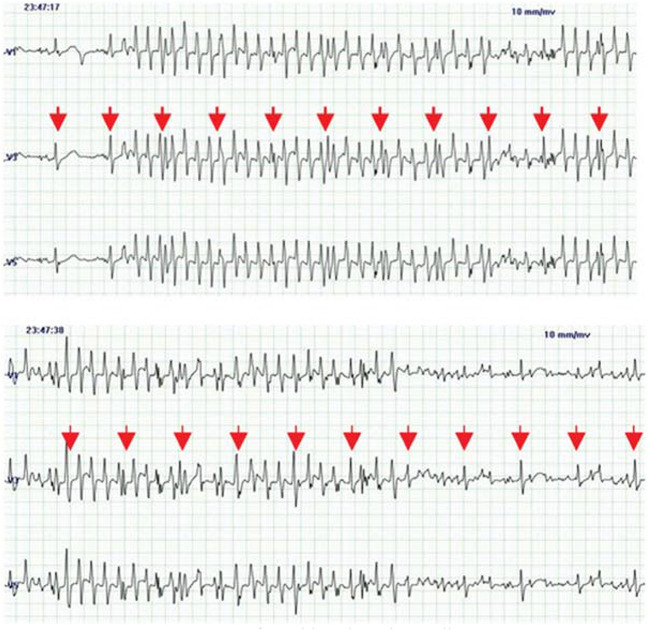
A case of toothbrush tachycardia [76]

Figure 7 demonstrates the influence of body movement on the recorded ECG signal. The first part exhibits baseline wander, which can be filtered out using a High Pass Filter (HPF) to obtain a clean signal. In the second part, the QRS complex is entirely obscured by MAs, but it can potentially be restored using state-of-the-art MA removal techniques. However, in the saturated area, the amplifier is overwhelmed, resulting in a signal that is entirely lost and cannot be reconstructed. This issue can result in physicians facing challenges in diagnosing various CVDs accurately, and in critical situations, it may lead to the misdiagnosis of heart conditions. Recently, a phenomenon known as "toothbrush tachycardia" has gained significant attention, as depicted in Fig. 8. The illustration explains a scenario where a patient with paroxysmal atrial fibrillation, who had initiated flecainide treatment three weeks prior, underwent a Holter ECG examination. The recording indicated two brief episodes of rapid heart activity, occurring before bedtime and in the morning. Upon further inquiry, it was discovered that these episodes coincided with the patient's toothbrushing routine. Currently, the only reliable method to confirm or refute this observation is through cross-referencing with the patient's diary, a detail that physicians may not always have readily available.

While MA is widely recognized as a significant drawback in many studies, certain researchers, such as Wang et al. [35] identify the main challenge in capacitive measurement systems as the high impedance created between the body and the electrode. To mitigate this issue, they focused on reducing this impedance by designing a negative impedance in series with the formed impedance. in another study, Zompanti et al. [77] considered CM signals generated by EMI as the only interferences, and they attempted to eliminate them by implementing DRL circuits and filtering techniques.

**Fig. 9:**
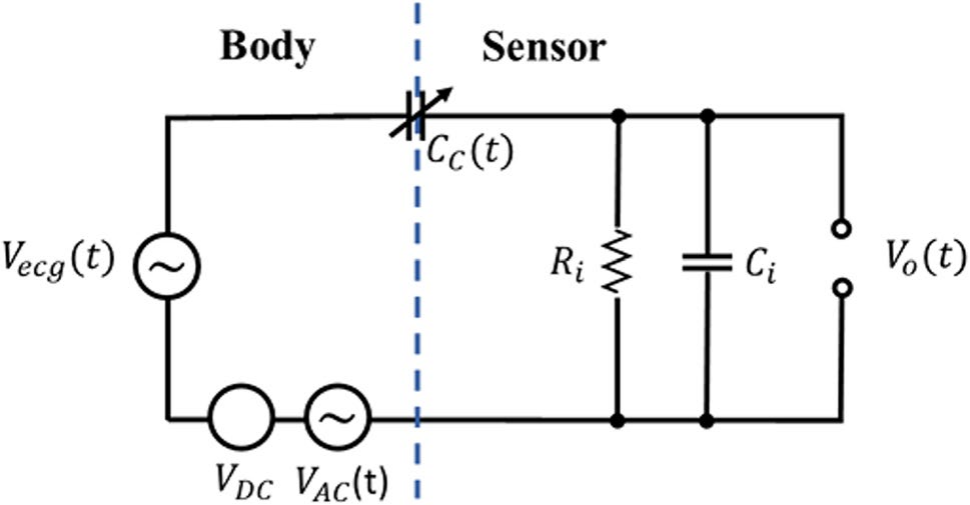
Electrical model of a single capacitive electrode

In many studies investigating and modelling MAs in capacitive measurement systems, researchers have explored an electrical equivalent model for a single capacitive electrode, akin to the one shown in Fig. 9 [12, 32, 38, 39, 70, 78].

In this model, the ECG signal is represented as ${V}_{ecg}(t)$, The input resistor and capacitor of the buffer are modelled as ${R}_{i}$ and ${C}_{i}$. Furthermore, ${C}_{C}(t)$ symbolizes the variable capacitor created between the body and the capacitive electrode. The leakage potential between the body's virtual ground and the reference point of the measurement system is determined by the sum of ${\text{V}}_{\text{DC}}$ and ${V}_{AC}(t)$. The voltage across the coupling capacitor can originate from various sources, leading to debates and disagreements about its origin. One contributing factor is triboelectricity [39], which results from the accumulation of static charges due to friction between the body and the electrode surface. The act of rubbing two materials together can produce significant voltages [12, 32, 39], although the exact underlying physics remains somewhat uncertain. A potential explanation involves an enlarged contact area due to rubbing, facilitating electron transfer between the materials. In addition to the effects of electrical double layers, other mechanisms contribute to contact voltage, such as the removal of charged particles or mass transfer between the two objects [79]. This phenomenon is also observable in classical ECG sensors, but it poses a greater challenge in capacitive sensors. This challenge stems from the prolonged negative impact resulting from the delayed discharge caused by the high impedance between the body and these sensors [12, 38, 80]. Consequently, this situation can lead to the generation of more substantial electric fields than those produced by the actual ECG signal [12].

A significant aspect originates from the skin potential, often referred to as electrodermal voltage [32, 39]. This voltage stands as a major contributor to MAs within the ECG signal; however, it has received limited attention, particularly in the capacitive measurement field. Comprehensive information concerning the nature and magnitude of this voltage remains elusive. Current research on wet electrodes reveals the presence of an inherent voltage source across the epidermis. This voltage has been observed to change due to skin deformation caused by lateral stretching, rotational stretching, or vertical forces [81, 82]. Notably, variations in impedance do not influence this voltage. The design of the electrode's structure significantly affects the degree of interference [83]. Despite its significance, there have been no studies to date that explore the fluctuations in skin potential in response to pressure or the motion of capacitive sensors. This knowledge gap emphasizes the necessity for additional research in this specific area.

DC bias currents from amplifiers can also contribute to this phenomenon [39]. An additional source of leakage potential comes in the form of PLI potential. Moreover, it can be related to earthing, also known as grounding, occurs when the human body comes into direct contact with the Earth, allowing for the free transfer of ions between the two. Recent research has highlighted a prevalent belief suggesting that the human body tends to maintain a positive charge, a phenomenon that cannot be effectively balanced due to the prevalent insulation from the Earth in modern lifestyles. In the past, routine activities such as walking barefoot and sleeping on the ground provided continual grounding, but today, the use of insulated footwear and the insulation of buildings from the ground have become commonplace [84]. Since these potentials contain a combination of both DC and AC voltages, they are represented as ${\text{V}}_{\text{DC}}+{V}_{AC}(t)$ in the model.

In the illustrated model shown in Fig. 9, the expected output is ${V}_{ecg}(t)$. However, the calculation of the current flow through a variable capacitor is expressed by Eq. (7) and this equation changes the characteristics of leakage potentials [32, 38, 39, 80].

7 $${I}_{c}=\frac{d\left(C(t)V(t)\right)}{dt}=C\left(t\right)\frac{dV(t)}{dt}+V\left(t\right)\frac{dC\left(t\right)}{dt}$$

The equivalent circuit of the capacitive sensor shown in Fig. 9 can be mathematically described in the time domain by a differential equation according to Kirchhoff's Current Law (KCL) as

8 $$\frac{d\left[\left({C}_{c}\left(t\right)+{C}_{i}\right).{v}_{out}\left(\text{t}\right)\right]}{dt}+\frac{{v}_{out}\left(\text{t}\right)}{{R}_{i}}=\frac{d\left[{C}_{c}\left(t\right).({v}_{ecg}\left(t\right)+{v}_{DC}+{v}_{AC}\left(t\right))\right]}{dt}$$

As a linear system, the output signal ${v}_{out}\left(\text{t}\right)$ is expected to comprise the superimposition of four components, which are the output due to ${v}_{ecg}\left(t\right)$, ${v}_{AC}\left(t\right)$, and ${\text{v}}_{\text{DC}}$ as shown in (9).

9 $${v}_{out}(\text{t})={v}_{ou{t}_{ecg}}+{v}_{ou{t}_{DC}}+{v}_{ou{t}_{AC}}$$

By using standard techniques for differential equations with time-varying coefficients we can solve (8) and obtain

10 $${v}_{ou{t}_{ecg}}=A(t){\int }_{0}^{t}\frac{d\left[{C}_{c}\left(u\right) .{v}_{ecg}\left(u\right)\right]}{du}{e}^{-{\int }_{u}^{t}f(s)ds}du$$

11 $${v}_{ou{t}_{DC}}={v}_{DC}.A(t){\int }_{0}^{t}\frac{d{C}_{c}\left(u\right)}{du}{e}^{-{\int }_{u}^{t}f(s)ds}du,$$

12 $${v}_{ou{t}_{AC}}=A(t){\int }_{0}^{t}\frac{d\left[{C}_{c}\left(u\right) .{v}_{AC}\left(u\right)\right]}{du}{e}^{-{\int }_{u}^{t}f(s)ds}du,$$

with:

13 $$A\left(t\right)=\frac{1}{{C}_{c}\left(t\right)+{C}_{i}}, f\left(t\right)= \frac{1}{{R}_{i}[{C}_{c}\left(t\right)+{C}_{i}]}$$

Reveal that each component in ${v}_{out}$ is a function of the coupling capacitance ${C}_{c}\left(t\right)$ and the corresponding source.

Equation (10) demonstrates the dynamic high-pass filter effect discussed in Section 3. It illustrates how the time-varying coupling impedance distorts the ECG signal [12]. This distortion converts ${v}_{ecg}(t)$ into ${v}_{ou{t}_{ecg}}(t)$, producing multiplicative noise [32], which is of lesser significance if the cut-off frequency of the high-pass filter in Eq. (4) is low enough.

The main challenge relates to Eqs. (11) and (12), where ${\text{V}}_{\text{DC}}$ and ${\text{V}}_{\text{AC}}$ undergo amplitude modulation due to ${C}_{c}\left(t\right)$. The changing coupling impedance transforms leakage voltages into an AC signal with an unknown frequency, producing additive noise [32] to ${v}_{ou{t}_{ecg}}$ which is shown in Eq. (9). Various studies have indicated that both intended and unintended body movements mostly occur within a frequency range below 10 Hz [32, 85, 86], although some have suggested a range from 0 to 20 Hz [38, 39, 59]. The chosen frequency range (0.1–20 Hz) covers breathing movements (0.1–2 Hz), body motion (0–10 Hz), and ballistocardiograph vibrations (0–20 Hz), aligning with the changing ${C}_{c}\left(t\right)$ [39]. As this range overlaps with the ECG signal, basic frequency-based filtering may not adequately eliminate AC-transformed voltages.

Additionally, the leakage voltages across ${C}_{c}\left(t\right)$ can reach several hundred millivolts or even a few volts, primarily depending on the static charge on the body surface or textile layers at the body-electrode junction. In the worst-case scenario, this voltage could entirely mask the measured biopotential and potentially cause system distortion. This transformation of mechanical vibrations into undesirable electrical signals is sometimes called microphonics [39], sharing similarities with signals resulting from membrane movements in microphones [36]. In summary, microphonics can modulate the amplitude of body surface potentials captured by capacitive electrodes, regardless of whether these potentials originate from bioelectric or triboelectric sources. This modulation reflects variations in the distance between electrode plates triggered by activities like breathing, heartbeats, and voluntary body motions [59]. In Eq. (14), $a\left(t\right)$ represents what is known as the MA (additive noise), regardless of the source of leakage voltages (${V}_{DC}+{V}_{AC}$). The signal s(t) represents the distorted version of the ECG signal in Eq. (15), contaminated with multiplicative noise. Consequently, $x\left(t\right)$ in Eq. (16) describe the recorded signal by the system.

14 $$a\left(t\right)={v}_{ou{t}_{DC}}+{v}_{ou{t}_{tri}}$$

15 $${s\left(t\right)= v}_{ou{t}_{ecg}}$$

16 $$x\left(t\right)=s(t)+a(t)$$

While these equations have been validated in numerous papers using the defined model, some studies suggest that the alteration in impedance may not be the sole cause of MA [87, 88]. Nevertheless, it is generally agreed that both electrode motion and changes in electrode–skin impedance contribute to MA [89].

**Fig. 10:**
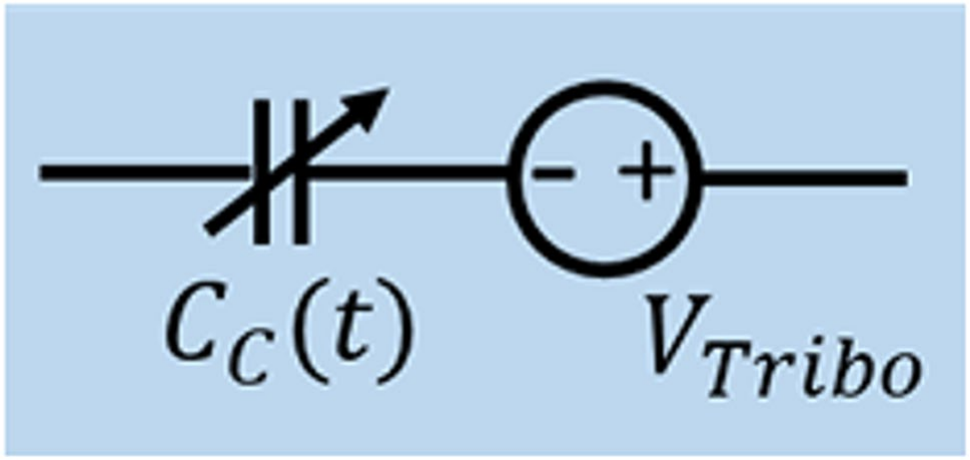
Electrical coupling of skin–electrode interface for a capacitive electrode

**Fig. 11:**
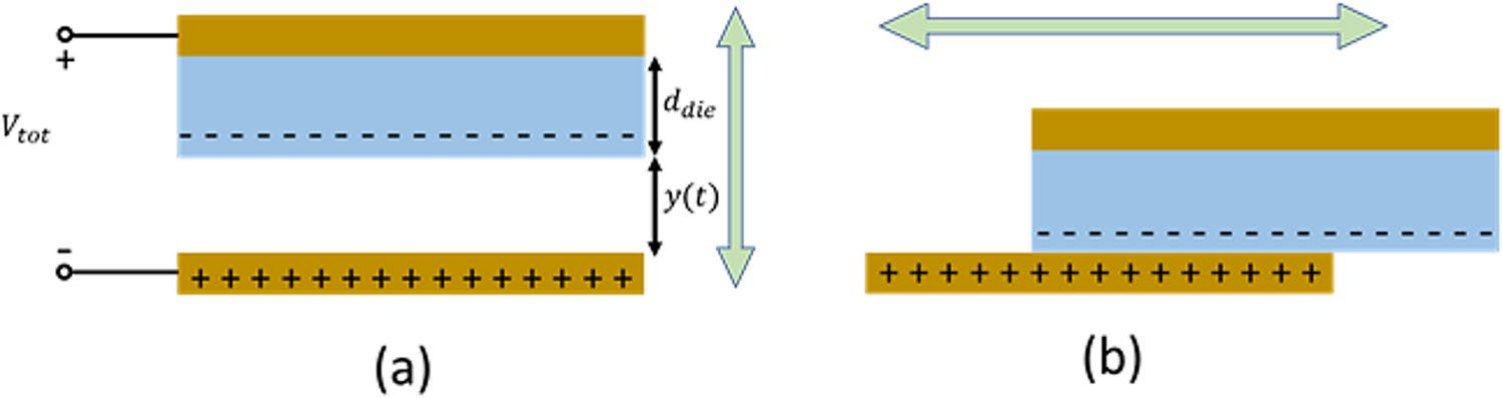
Fundamental modes of TENGs. **a** Transversal contact-separation mode. **b** Lateral mode

In another existing category, MA is divided into two sectors: MA caused by transversal and lateral electrode movements. Transversal movement brings about changes in coupling distance, which alters coupling capacitance and gives rise to the microphonic effect. On the other hand, lateral movement creates friction at the interface between the body and the electrode, resulting in triboelectricity [30, 36, 70, 79]. Nevertheless, this explanation might not cover all aspects. Transversal movement, alongside lateral movement (sliding and friction), can also generate triboelectricity. This is especially true when using rigid PCBs as electrodes that may not align perfectly with the body's surface. In such cases, significant changes in the gap between the PCB and the body occur during motion [90]. Furthermore, when two surfaces previously in contact separate from each other, there is charge accumulation and redistribution due to triboelectricity. This leads to a significant amount of noise being introduced and interfering with the desired signal. However, lateral motion produces even greater friction and triboelectricity. This phenomenon is like what happens in triboelectric nanogenerators (TENGs). In this model, the coupling between the electrode and skin consists of not only a capacitor $\left(C_c\left(t\right)\right)$ but also includes a voltage source $\left(V_{Tribo}\right)$, connected in a series, as illustrated in Fig. 10 [30, 91]. TENGs operate based on the triboelectric effect and displacement current principle, similar to capacitive electrodes. They have different operational modes with specific structures, among which the transversal contact-separation mode (Fig. 11. a) and lateral mode (Fig. 11. b) exhibit a triboelectric effect like that of capacitive electrodes.

A model explaining the triboelectric effect on capacitive electrodes can be derived from the V-Q-x relationship of TENGs, as shown in Eq. (17). Sirtoli et al. [30] formulated this model for situations involving contact-separation, as expressed in Eq. (20). The derivation of this equation involves calculating ${C}_{c}\left(y\right)$ using Eq. (18), where $y(t)$ signifies the time-variable distance between the electrode and the body surface. Additionally, the calculation of ${V}_{Tribo}(y)$ is carried out using Eq. (19). ${\sigma }_{d}$ represents the surface charge density, y(t) refers to the displacement at a given time (t), ${\varepsilon }_{die}$ is the dielectric permittivity of the capacitive electrode, and ${\varepsilon }_{0}$ stands for the vacuum permittivity.

17 $${V}_{tot}=-\frac{1}{{C}_{c}\left(y\right)}Q+{V}_{Tribo}\left(y\right)$$

18 $${C}_{c}(y)=\frac{A{\varepsilon }_{0}}{\frac{{d}_{die}}{{\varepsilon }_{die}}+y(t)}$$

19 $${V}_{Tribo}=\frac{{\sigma }_{d}y\left(t\right)}{{\varepsilon }_{0}}$$

20 $${V}_{tot}=-\frac{Q}{A{\varepsilon }_{0}}\left[\frac{{d}_{die}}{{\varepsilon }_{die}}+y\left(t\right)\right]+\frac{{\sigma }_{d}y\left(t\right)}{{\varepsilon }_{0}}$$

## The latest techniques for MAs reduction

To minimize the disturbance from MA, it's crucial to address three key areas: Analog circuits (dealing with electrical components), Materials and Fabrication (selecting suitable electrode materials, and optimizing fabrication) and DSP approaches. These elements must collaborate to efficiently reduce noise and improve the overall quality of the ECG signal.

### Analog circuits

The circuit design techniques in Section 3 can help reduce MA by mitigating leakage potentials. Therefore, addressing this section is highly necessary. However, when measuring ECG with two active electrodes, a challenge arises as these electrodes can move independently, causing varying MAs. Different frequency changes of ${C}_{c}\left(t\right)$ and leakage voltages (${\text{V}}_{\text{DC}}+{V}_{AC}(t)$) for each electrode lead to different additive MA, (a(t) in Eq. (14)). Additionally, the triboelectric voltage from Eq. (20). varies due to the varying distance between the electrode and the body (y(t)). Consequently, the CMRR factor cancels out extra CM voltages, except for the portion transformed into a differential signal due to the skin–electrode impedance mismatch. This transformed portion is then amplified by the differential mode gain of the differential amplifier, degrading the output signal [64]. Enhancing CMRR through the mentioned techniques in Section 3 does not offer a solution to this issue.

### Materials and fabrication

**Fig. 12:**
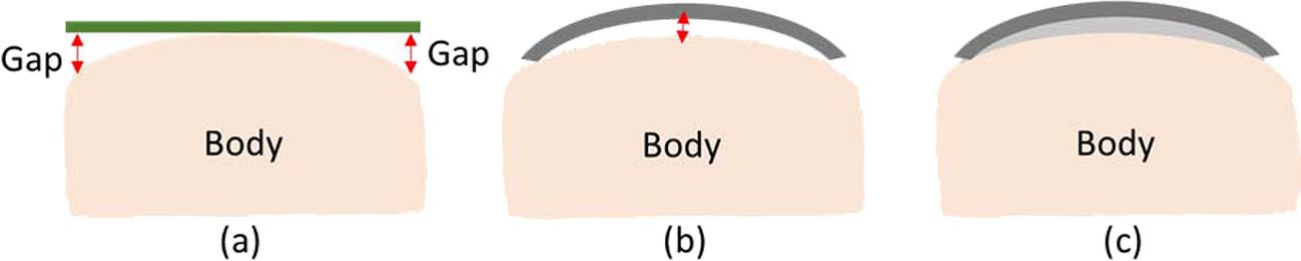
**a** Rigid electrode. **b** flexible electrode. **c** flexible electrode with conductive foam

Research in another area examines the characteristics of capacitive electrodes—size, shape, and material—that affect system performance. Expanding the surface area is beneficial for increasing coupling capacitance. However, rigid materials have limitations in surface expansion, as shown in Fig. 12. (a), due to difficulties in full adherence to the body. Even with added layers of cotton or cloth, gaps persist, impacting impedance and signal quality [21]. To overcome this, researchers are investigating flexible electrodes that can conform to the chest's curvature, as depicted in Fig. 12. (b). This improves contact surface and, consequently, enhances coupling capacitance [21, 90]. Nevertheless, Lee et al. [92] suggested that when conventional flexible electrodes are used, air gaps can still exist due to the chest's uneven surface caused by ribs. To compensate for this, they propose the use of conductive foam, as depicted in Fig. 12. (c), to eliminate additional air gaps. Moreover, Asl et al. [28] proposed that by increasing capacitance with flexible electrodes, we can use a lower bias resistor to achieve a low enough ${f}_{c}$ in Eq. (4), thereby minimizing voltage noise. Importantly, prior research [28] indicates that the electrode shape (circular, rectangular, etc.) has no effect on voltage noise. Additionally, High-spatial resolution ECG recordings provide valuable insights for diagnosing a wide array of cardiac abnormalities, including infarction and arrhythmia [93]. To enhance spatial resolution in ECG, researchers have developed body surface potential maps by expanding the standard 12-lead ECG to encompass a greater number of recording points across the body surface [94]. It is widely recognized that reducing the spacing between electrodes leads to higher spatial resolution [93]. Additionally, it is important to consider the impact of electrode size on spatial resolution. Studies have shown that enlarging the area of the plate electrode reduces spatial resolution [95]. While our discussion primarily focuses on capacitive electrodes with fewer leads, it is essential to consider the trade-off between increasing the surface area to enhance capacitance for improving signal strength and preserving spatial resolution. This balance requires further exploration.

Numerous research studies have explored diverse dielectric materials and their influence on bioelectrode performance. A higher dielectric constant enhances capacitance, but the intended application must be carefully considered. Metal oxides, for example, provide a high dielectric constant suitable for capacitive biosensing; however, their reactivity with sweat may lead to more MAs [20]. Moreover, fabrics and polymers are typically characterized through chemical processes, making it essential to consider their reactivity with skin exudates [20]. Capacitive electrodes with insulated layers are designed to minimize skin reactions, ensure stable interfaces, and enhance overall patient comfort [21]. However, the selection of unsuitable materials can impede the achievement of these goals. For instance, using anodic aluminum oxide ($A{l}_{2}{O}_{3}$) as the dielectric can lead to interactions with sweat chloride ions, causing discomfort for patients [96]. Furthermore, as stated earlier, friction and the separation of dielectric surfaces can produce charges against the skin, ultimately leading to MA. In such scenarios, two fundamental principles are employed to assess the quantity of generated charge from a dielectric material on a surface, aiding in the selection of materials with reduced charge generation. The first principle is “charge affinity”, which classifies materials based on their inclination to exchange electrons through contact/friction. The second is TriboElectric Charge Density (TECD), a measure of the interaction between two materials influenced by factors such as humidity, surface roughness, temperature, stress, and other mechanical properties [30, 91].

### DSP approaches

**Fig. 13:**
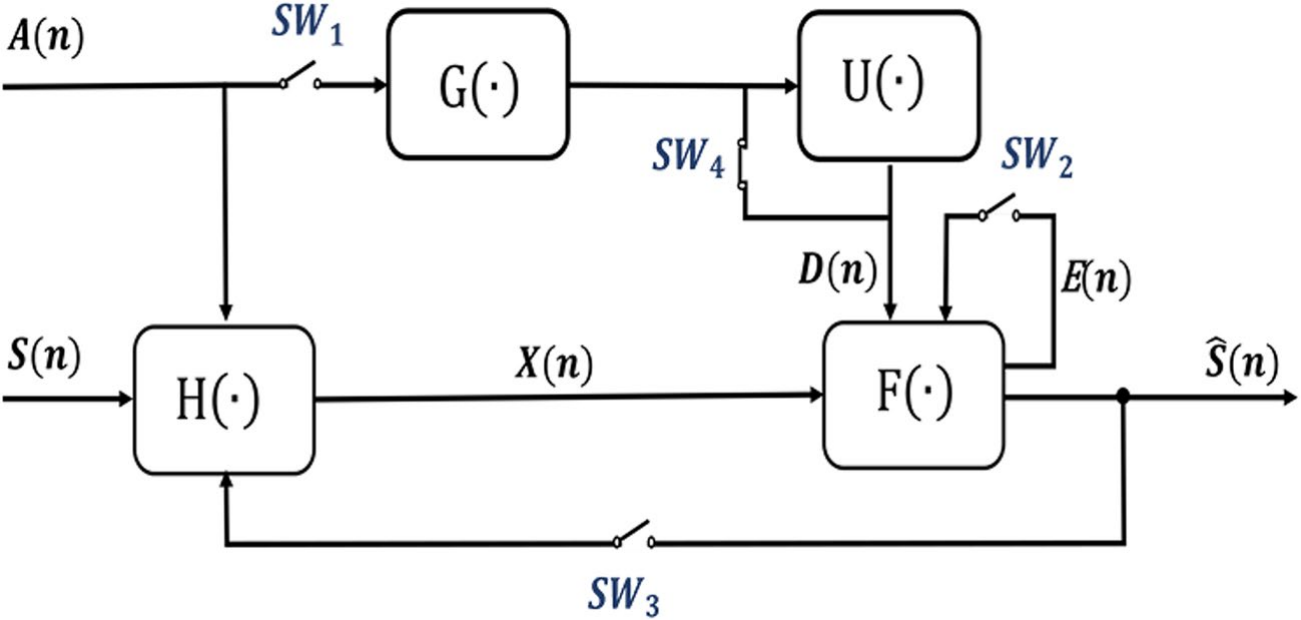
A general framework for artifact removal in DSP methods

After addressing interference in the two previous sections, any remaining issues can be tackled within the DSP domain. DSP is an integral component of ECG monitoring systems. Figure 13 presents a general overview of the multi-channel artifact suppression techniques discussed in this review, inspired by previous articles [21, 30, 97]. Three matrices — $\text{S}\left(\text{n}\right)$, $\text{A}(\text{n})$, and $\text{X}(\text{n})$ — comprise multiple column vectors of signals. Each rectangular block symbolizes a generalized transfer function, such as linear time-variant, nonlinear time-invariant functions, etc. The process involves combining two signals: $\text{S}\left(\text{n}\right)$ (uncontaminated ECG signal) and $\text{A}(\text{n})$ (MAs), using a transfer function denoted as $\text{H}\left(\cdot\right)$. The outcome is the final recorded signal, $\text{X}(\text{n})$, which contains both multiplicative and additive MAs. $\text{X}(\text{n})$ represents Eq. (16) discussed in Section 4. Triboelectric potential outlined in Eq. (20) can also be integrated into this signal. This diagram can also incorporate future modifications to the MA model.

Various filtering methods can be conducted through the filtering subsystem, represented as operator $\text{F}\left(\cdot\right)$. Some studies employ high-pass or band-pass filters, represented by opening switches $S{W}_{1}-S{W}_{4}$ in Fig. 13, to eliminate MA. However, these approaches are rudimentary and can introduce further signal distortion due to the overlap of MA frequencies with the ECG signal, as well as similarities in shape to P and T waves [74, 98]. By utilizing switches $S{W}_{1}$—$S{W}_{4}$, optional paths and blocks can be employed. The configuration and positioning of these elements differ across various systems. Instead of selecting a straightforward filtration process, a different approach was taken by Ottenbacher and Heuer [70]. They investigated restoring the ECG signal by solving the inverse system function, $\text{H}^{-1}\left(\cdot\right)$, and utilizing it as $\text{F}\left(\cdot\right)$. However, their reconstruction technique demands knowing all model parameters, a challenge that is frequently hard to meet.

Consequently, many studies have turned to computationally intensive algorithms for suppressing MAs. These approaches can be categorized into two groups: self-referencing techniques that do not require additional signals, and therefore, do not necessitate the use of $\text{G}\left(\cdot\right)$ and $\text{U}\left(\cdot\right)$ (indicated by the open switches $S{W}_{1}$-$S{W}_{3}$), and those that necessitate supplementary signals for signal analysis ($S{W}_{1}$-$S{W}_{2}$ are closed, while $S{W}_{3}$-$S{W}_{4}$ are optional).

#### First category: self-referencing techniques

The first category exclusively relies on DSP techniques to filter out MA. More traditional methods, such as filter banks [99], use time averaging and frequency analysis, assuming the statistical independence of MA and the signal, which has not been verified and can distort the original ECG signal to some extent [100].

On the other hand, the wavelet transform is widely used as a denoising method due to its time–frequency localization and multi-resolution characteristics [101–104]. This method can effectively describe the non-stationary characteristics of the signals, such as ECG. This method has three steps: decomposition, denoising, and reconstruction. First, the original signal is decomposed into an approximate coefficient and several levels of detail coefficients using low-pass and high-pass decomposition filters. Then, all the decomposed coefficients are processed with a denoise algorithm, and the inverse discrete wavelet is applied to reconstruct a noise-free signal [78]. However, the choice of wavelet is important for denoising performance, and comparative studies have shown different conclusions mainly due to differences in the noise characteristics used. The wavelet transform is also computationally intensive and depends on specific MA and ECG signal characteristics and parameters, which may limit its effectiveness. Additionally, since MA is time-varying, wavelet transforms may not be effective in separating the artifact from the ECG signal [75].

Blind Source Separation (BSS) techniques assume that the sources of ECG and MA are uncorrelated and require input ECG signals to be linearly independent from each other [105, 106]. Although these methods are popular and widely used, they require many recording channels and high computational power due to their high algorithm complexity for good performance. Additionally, BSS methods are blind to the wider sensing situation and assume that all ECG channel signals are affected by the same source of noise, which is implausible due to the spatial distribution of skin impedance under the electrode sensors [98, 107].

According to the literature, these methods are not effective enough, and MA remains largely in the system output [75]. They are also unsuitable for wearable applications that require a low number of parallel channels, small size, and quick setup, while the available processing power is limited by power consumption constraints [107].

#### Second category: reference-assisted techniques

**Fig. 14:**
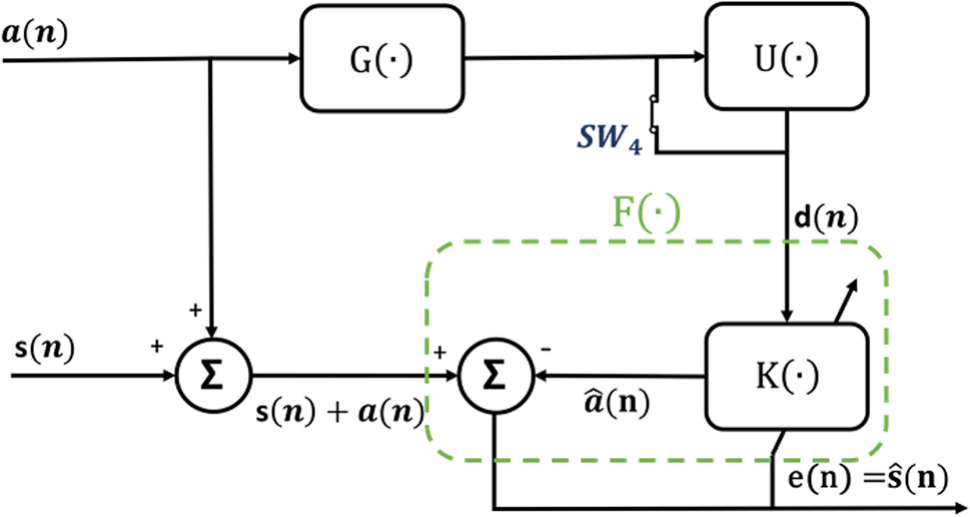
A general framework for artifact removal for reference-assisted techniques

Figure 14 provides a more detailed illustration of the second category, derived from Fig. 13. In this representation, $\text{H}\left(\cdot\right)$ is simplified as a sum block. Consequently, the output signal, $\text{X}(\text{n})$, results from the summation of the signal of interest, $s\left(n\right)$, and the MA, a(n). The operator $\text{G}\left(\cdot\right)$ denotes a measurement subsystem generates a reference signal, $d\left(n\right)$ for the input of $\text{K}\left(\cdot\right)$. $\text{K}\left(\cdot\right)$ generates $\widehat{a}(n)$, an estimation of the MA, $a(n)$. Subsequently, $\widehat{a}(n)$ is subtracted from the recorded signal.

Several assumptions are associated with this method:

1. The MA is not correlated with the signal of interest, $s\left(n\right)$.
2. The reference signal is correlated with the MA, $a\left(n\right)$.
3. The reference signal is not correlated with the signal of interest, $s\left(n\right)$.

The difference between the main input signal, $x\left(n\right)$, and the output, $\widehat{a}\left(n\right)$, of the noise reduction system can be utilized as the error output, represented as $e\left(n\right)$, as shown in Eq. (21):

21 $$e(n)=s(n)+a(n)-\widehat{a}(n)$$

The mean square of $e\left(n\right)$ is given by Eq. (22):

22 $$E\left[{e}^{2}\right]=E\left[{\left(s+a-\widehat{a}\right)}^{2}\right]=E\left[{s}^{2}\right]+E\left[{\left(a-\widehat{a}\right)}^{2}\right]+2 E\left[s(a-\widehat{a})\right]$$

If $s(n)$ is not correlated with $d(n)$, Eq. (22) simplifies to Eq. (23):

23 $$E\left[{e}^{2}\right]=E\left[{s}^{2}\right]+E\left[{\left(a-\widehat{a}\right)}^{2}\right]$$

As the difference between $a(n)$ and $\widehat{a}$(n) approaches zero, the error output $e$(n) can be considered the best least-squares estimate of the ECG signal, as shown in Eq. (24). The effectiveness of noise reduction relies on the level of correlation between $d$(n) and $a(n)$. Therefore, ensuring a high correlation between them is essential to suppress the noise [108].

24 $$e(n) \approx s(n)$$

This method can be categorized into two types. In the first type, there is an assumption that $\text{K}\left(\cdot\right)$ represents a known transfer function for estimating MA. In the second type, an adaptive noise filtering approach is employed to estimate MA. In this scenario, the correlation between the reference signal and MA becomes crucial.

### First type: $K\left(\cdot\right)$ is known

In the first type, the entire system is mathematically modeled, where $\text{K}\left(\cdot\right)$ represents a known transfer function containing certain unspecified parameters. Unspecified parameters in this method can be estimated using the reference signal and Block least-squares minimization. Equation (22) is then employed to mitigate MA. This technique is highly effective in eliminating MA if the model is accurately calculated; otherwise, it may not yield acceptable results. Serteyn et al. [39] utilized this method, demonstrating effectiveness in both simulation and lab setups. However, the proposed method does not completely eliminate the artifact under real-life conditions. Factors such as a simplified system model, thermal noise, and other artifacts like triboelectric effects and EMI induced by unaddressed motion may obscure R-peaks.

### Second type: adaptive noise filtering

Active noise cancellation [109] or adaptive noise filtering [74] methods estimate the transfer function, $\text{K}\left(\cdot\right)$, between a reference signal and the MAs [32]. Essentially, by employing a reference signal that correlates with the MA present in the recorded ECG, the adaptive filter adjusts its coefficients to minimize the error signal, $e\left(n\right)$, as defined in Eq. (23). This adjustment aligns the reference signal with the MA [40]. The recorded signal is subtracted from the mapped reference signal, resulting in an output with cleaner ECG waveforms [74]. This approach shows promise in MA removal due to its low computational complexity.

The coefficients of this nonlinear filter frequently change based on predefined conditions [106]. In cases where the characteristics of MAs change over time due to movement, adaptive filtering proves to be more effective than other methods. Its ability to adapt parameters facilitates better capture of evolving signal characteristics. While adaptive filtering algorithms can be implemented with a single-channel input, achieving high filtering performance necessitates a reference signal that exhibits a strong correlation with the additive noise [32, 100, 105]. The limited correlation between the two signals often leads to more contamination of the desired ECG signal [74]. Moreover, alongside recording the ECG, simultaneous recording of a reference signal is essential [106].

The adaptive filter can be implemented using a variety of adaptive algorithms, with the most commonly employed ones being Least Mean Square (LMS), Normalized Least Mean Square (NLMS), and Recursive Least Square (RLS) [32, 74, 75, 105]. Each of these approaches possesses its own set of advantages and disadvantages.

Many forms of reference signals, $d(n)$, have been employed for MA suppression, such as the measurement of skin stretching with optical sensors [110, 111], electrode motion using accelerometers [112, 113], deformation of electrodes [114], and the change in ETI [32, 39, 40, 74, 106, 109, 115, 116].

In a recent study, Ding et al. [108] introduced a novel reference signal using custom electrodes made of silver conductive fabric. Each electrode contained three layers: a rectangular layer of conductive material for acquiring ECG signals, a rectangular insulation layer above the previous layer, and a comb-tooth-shaped layer for acquiring the reference signal. This method showed good results; however, it adds one more signal acquisition channel. The main limitation of second category DSP methods is that they require additional sensors to measure a reference signal that correlates with motion and must be synchronized with the capacitive sensor [32, 39]. This solution may increase the complexity and power consumption of the recording system, and in some cases, their method is not automated [39], making it unsuitable for ambulatory monitoring, which is the primary goal of capacitive ECG measurements [32].

Among the mentioned reference signals, ETI seems a promising candidate. Although the first suggestion was to use an auxiliary capacitive sensor to track changes in the coupling impedance [117], other studies have shown better solutions that do not require additional sensors. ETI is a signal of significant interest since it exhibits a high correlation with MA, allowing for superior performance of beat detection algorithms compared to the use of other reference signals. Additionally, it is amenable to low-power circuit design since no additional sensor is required to produce ETI [74, 106]. The ETI signal can be recorded continuously and simultaneously with the ECG signal by sharing electrodes, injecting higher-frequency AC signals into the sensing electrodes, and amplifying the resulting voltage [40, 105]. Since the injected AC signals have spectral contents outside the ECG band, they do not corrupt the ECG's morphology [38, 74]. This technique is also utilized in traditional ECG measurement systems employing gel or dry electrodes, but it has also been recently suggested for use in ECG measurement systems employing capacitively coupled electrodes [106]. In recent studies on capacitive electrodes, an injection signal has been employed for tasks such as channel selection [118], respiration observation, artifact localization [59], and impedance measurement in static conditions [119]. However, its use for artifact reduction or ECG reconstruction had not been explored until Serteyn et al. [120] conducted the first test in this regard. Eilebrecht et al. [119] then measured the static value of the coupling capacitance for different textiles and electrode designs. Buxi et al. [106] used an injection current at 20 kHz to monitor the ETI of wet and dry electrodes for different MAs. Torfs et al. [40] used a Howland current pump with very small current ranges to inject to the electrodes. In this measurement setup, the first indications of periodic MAs were seen, which could have been associated with heart activity. Current injection has been proposed for many wet and dry electrodes, too [105, 115, 116, 121]. Instead of injecting a current, another adaptive filtering approach injected voltage into capacitive electrodes to measure the ETI [39, 120, 122]. Demodulating the PLI allows for extracting a reference signal that reflects variations in coupling capacitance for adaptive MA removal, as proposed by Xu. et al. [32]. They conclude that the reference signal obtained by this technique can include components that reflect variation in the PLI amplitude in addition to the variation in the coupling impedance. This can lead to an overestimation or underestimation of the MAs.

One important challenge is measuring the ETI signal without impacting the quality of biopotential signals and minimizing crosstalk [115, 123].

### Challenges of MA reduction using adaptive filtering with ETI as the reference signal

Among the studied methods for reducing MA, the combination of adaptive filtering with an ETI reference signal has received significant attention. However, literature highlights that unlike the clear and direct relationship between the impedance signal and motion pattern, the correlation between MA and ETI signals is not often strong and straightforward. As a result, efficiently subtracting MA becomes challenging [82]. Buxi et al. [106] investigated the relationship between MA and ETI for different artifacts, types of electrodes, and impedance signals. Their goal was to understand when ETI could effectively assist in reducing MA through algorithms. Their findings indicated that among the chosen electrodes, local electrode artifacts (such as pushing and pulling the electrode) had the highest correlation with MA. This was followed by local skin artifacts (like stretching, twist, skin), and then global artifacts (including actions like walking, jogging, jumping). Therefore, it becomes essential to understand the cause or mechanism of MA in order to identify a reference signal that is strongly connected to MA [100]. The ETI signal is usually thought to be purely capacitive in capacitive ECG measurement. Even materials like cotton or solder masks, which insulate well, show resistance in the range of a few Giga ohms. This is significantly lower than the input impedance of a common operational amplifier. Moreover, these materials exhibit some resistive characteristics within specific ECG frequency ranges [124]. Apart from the fully capacitive model, there are other impedance models of the ETI. One proposed by Akabane et al. [125], involves using a combination of a series resistor and a parallel arrangement of resistor and capacitor. Buxi et al. [106] studied the Bode-plot of the skin–electrode impedance. They introduced three different components (real part, imaginary part, and absolute impedance) to provide a comprehensive description of the impedance signal. Addressing the issue of limited correlation, Cömert and Hyttinen [82] focused on the frequencies used for impedance measurements. They noticed that impedance responses at various frequencies differed in the waveform when subjected to the same motion. In the realm of studying motion, higher-frequency impedance measurements displayed a stronger connection to motion compared to lower frequencies. At higher frequencies, impedance measurements turned out to be more sensitive to minor movements, thus delivering more accurate evaluations of applied motion across different motion levels. Additionally, these higher impedance frequencies could act as indicators of applied motion. They showcased that even though the correlation coefficients might be lower, higher frequencies exhibited stability in their relationship to the MA signal both in terms of time and frequency. They concluded that while impedance might not directly predict the shape of the MA signal over time, it could anticipate the key frequency components of the MA signal. Furthermore, it serves as an effective predictor of the actual applied motion.

To facilitate discussion of the remaining aspects, a comparative table is employed to offer a comprehensive overview of the previous works, as depicted in Table 3. Serteyn et al. [39] applied the Block least-squares parameter estimation method to real-life data, achieving a reduction in artifact RMS amplitude by 9 dB. However, they noted instances of unclear visibility of R-peaks in certain sections. They suggested that remaining artifacts might stem from factors not addressed in their study or arise from the simplified electrical model they employed. In contrast, Torfs et al. [40] employed an LMS adaptive filter, resulting in an average reduction of 49% in the RMS content of the signal. They observed variations in the effectiveness of the capacitance impedance signal in correlation with different types of MAs. This finding highlights the importance of developing more sophisticated algorithms to fully take advantage of information from the capacitance impedance signal for effective MA reduction. In a similar study, Xu et al. [32] attempted to demodulate the PLI signal instead of injecting a signal to estimate ETI. They used an NLMS adaptive filter to mitigate MAs and claimed that their proposed method improved the system's SNR by an average of 1.2 dB, resulting in clearer visibility of R-peaks. However, they still observed high-amplitude MAs. They concluded that the reference signal obtained by this technique may encompass components reflecting variations in PLI amplitude, as well as variations in coupling impedance. This dual effect could potentially lead to an overestimation or underestimation of MAs. In the following discussion of ETI as the reference signal, He and Min [100] suggested that the ETI signal may not exhibit the highest correlation with the MA. As a result, they generated a novel synthesized reference signal. This synthesized signal combines skin-induced impedance behavior, skin "slope," and recovery "time constant" to effectively mitigate MA. To address this, an additional processing step, denoted as the $\text{U}\left(\cdot\right)$ block in Fig. 14 was introduced. This step is designed specifically to enhance the correlation of the signal. In this scenario, the $S{W}_{4}$ component should be opened. Moreover, they addressed a key limitation of traditional LMS algorithms – the challenge of balancing convergence speed with convergence error. To overcome this trade-off, they introduced an enhanced LMS filter that incorporates both fast tracking and low convergence error components. This modification successfully navigated the delicate balance between speed and accuracy, yielding promising outcomes and be able to 7∼15 dB improvement on average in their system. But their experimental setup involved offline signal processing to adjust applied vertical forces to sensors, and they did not explore various artifact types in their study of MA. Recognizing the low correlation between MA and ETI, Pholpoke et al. [74] used an additional layer of digital algorithms, BSS, in the backend to effectively reduce MA. They also highlighted the need for recording devices with higher input dynamic ranges (DR) to handle MAs. This requirement necessitates existing systems to use low gain in their amplification chains to prevent output saturation. To tackle this challenge, they implemented LMS filtering to generate a cancellation signal capable of nullifying MA near the system's input. For this design $S{W}_{3}$ in Fig. 13 needs to be closed. By effectively suppressing MA, the signal could then undergo additional amplification without encountering output range saturation. In the field of using a more efficient method to tackle with different types of MA, Kim et al. [126] proposed a MA removal method employing more efficient techniques to address various types of muscle artifacts (MAs). Their approach utilized a two-stage cascade LMS adaptive filter. In the first stage, an LMS algorithm with analog feedback was employed to prevent signal saturation, similar to the technique used by Pholpoke et al. [74]. This aids in reducing the input dynamic range. For the second stage, an adaptive step-size LMS algorithm was used. This adaptive step-size algorithm facilitates rapid convergence to track large, sudden MAs while preserving the integrity of the ECG component. The filtering performance was evaluated based on heartbeat detection, measured by sensitivity (Se) and positive predictive value (+ p), resulting in an improvement of 9.8% and 6.48% respectively.

**Table 3:** Comparison of previous works

Source	Methods for measuring ETI	Methods of MA mitigation	Type of experiments	Results
Serteyn et al. [39]	Voltage injection at a frequency of 1 kHz	• Reverse the transfer function • Block least-squares parameter estimation	• Simulation • Lab Data • Real-Life Data ○ Electrode Pushing	• Good results in simulation and lab experiments ○ Achieved clear visibility of R-peaks • Real-life data: ○ Reduced artifact RMS amplitude by 9 dB ○ Observed unclear visibility of R-peaks
Torfs et al. [40]	Current injection at a frequency of 10 kHz	• LMS adaptive filter	• Real-Life Data ○ Electrode pushing/pulling and lateral motion ○ Arm motion	• Acceptable results in push/pull motion • Not acceptable results in Arm motion • Reduced RMS content of the signal by an average of 49%
Xu et al. [32]	Demodulation of the PLI signal	• NLMS adaptive filter	• In-silico experiment • In-vivo experiment ○ Subject seated on a chair with their trunk straight ○ Subject seated on the chair, oscillating their trunk ± 15 degrees forward and backward	• Acceptable results in in-silico experiment • In-vivo experiment: ○ High-amplitude MAs observed ○ Achieved clear visibility of R-peaks ○ 1.1 dB improvement in SNR for the first test ○ 1.3 dB improvement in SNR for the second test
He and Min [100]	Current injection at a frequency of 5 kHz	• Improved LMS filter ○ Balances speed and accuracy	• Real-Life Data ○ Electrode pushing/relaxing	• 7∼15 dB improvement on average (Performance metrics was not mentioned)
Pholpoke et al. [74]	Current injection at a frequency of 5 kHz	• Two stage MA mitigation ○ 1st-order lowpass filter ○ Sign-sign LMS adaptive filter	• Real-Life Data ○ Electrode pushing	• ––-
Kim et al. [126]	No information capable as they used database	• Two stage MA mitigation ○ LMS adaptive filter feedback to analog circuit ○ Adaptive step-size LMS adaptive filter	• Imec database	• Evaluated performance via heartbeat detection: ○ 9.8% improvement in sensitivity (Se) ○ 6.48% improvement in positive predictive value (+ p)

## Discussion and future directions

In capacitive ECG data loggers, it is evident that suboptimal circuit designs can introduce signal distortion, often misidentified as MAs. To address this issue, it is essential to carefully examine electrode construction and circuit design aspects, often overlooked in current literature. Rectifying these shortcomings will pave the way for a more comprehensive understanding of the origins of MAs. Presently, the definition and sources of MAs remain unclear. While there have been discussions on the study of MAs involving wet electrodes by introducing various artifacts to establish improved models, research efforts have predominantly centered around capacitive electrodes, given their growing demand in wearable telehealth monitoring systems. Consequently, most published works explore MAs, examining their amplitude and frequency characteristics across various applied artifacts in diverse ECG experiments utilizing capacitive electrodes. The ultimate goal is to present a reliable model for MAs.

Another related domain necessitating attention is DSP for MA removal. Although adaptive filtering, often utilizing the ETI as a reference signal, holds promise in mitigating this issue, it has yet to yield sufficient outcomes. Therefore, a more in-depth exploration of ETI is imperative and could serve as a main point for future research. Beyond solely relying on the capacitive model for skin–electrode impedance, there is room for investigating alternative models. Moreover, the circuit used for estimating the ETI requires further examination.

The efficacy of MA estimation through adaptive filtering significantly depends on the availability of a highly correlated "reference signal". While ETI is a widely accepted candidate, enhancing its correlation through pre-processing algorithms holds the potential for generating a more efficient reference signal. This approach has been adopted in studies involving wet and dry electrodes but remains unexplored in the context of capacitive electrodes. Additionally, using supplementary reference signals, such as accelerometer data, may hold promise for improving the SNR, a direction that merits investigation.

Another promising path for further research lies in the design of adaptive filtering itself. While current literature primarily concentrates on identifying reference signals that exhibit high correlation with MAs, designing adaptive filters capable of effectively mitigating MAs has received limited attention and deserves more research. Lastly Moreover, there are instances where combining DSP techniques with analog designs can potentially prevent amplifier saturation and signal loss, presenting another attractive area for future research.

It should be noted that besides traditional denoising techniques, there are other emerging approaches, such as machine learning and deep learning techniques, which are not as commonly cited in academic works. While deep learning techniques share certain characteristics with adaptive filters in the domain of ECG signal processing, they have primarily been utilized for tasks such as feature extraction and noise detection rather than direct ECG signal reconstruction. Although less frequently mentioned in the literature compared to traditional denoising techniques, the relevance of machine learning and deep learning methods in ECG denoising may increase in the coming years, particularly as research in this area continues to develop.

## Conclusion

This review highlighted the significance of capacitive ECG measurement systems as a promising alternative for ECG measurement, offering non-intrusive long-term monitoring. However, the challenge lies in maintaining diagnostic accuracy comparable to hospital-grade systems. MAs have emerged as a critical issue in capacitive ECG measurement due to the variable impedance between the body and the electrode, along with existing interference sources. Despite being commonly acknowledged; the exact origins and definitions of MAs remain unclear. Addressing this gap, we reviewed existing models. Furthermore, this article studied state-of-the-art MA mitigation techniques, spanning analog circuits, electrode fabrication, and DSP methods. Emphasis has been placed on adaptive filtering using ETI as a reference signal, consistent with the approach of many recent articles. While advancements have been made in this field, challenges persist, warranting further research in several areas concurrently. These include synthesizing a reference signal more correlated with MAs using ETI or add other reference signals, optimizing mitigation methods for improved accuracy and speed, developing advanced techniques adaptable to various MAs, and enhancing the dynamic range of the system to enable higher gains, as evidenced in prior studies.
